# Cold Baths

**Published:** 1876-07

**Authors:** 


*TWENTY-THIRD YEAR OF*]

### HALL'S JOURNAL OF HEALTH.

***E. H. Gibbs, M.D., Editor.***

VOL. XXIII.] JULY, 1876. [NO. 7.

### *COLD BATHS.*

Of all the popular fallacies, the most mischievous and the most difficult to overcome, is that which claims that cold baths promote health under all circumstances. Letters asking medical advice usually give particulars as to the mode of life and habits of the patient, and almost every letter informs us that the "cold bath" is not neglected. If it were, in very many cases, there would be no occasion for consulting a physician. Complete restoration to health has frequently followed a change in the temperature of the baths. The temperature of the blood, in health, is about 98 F. Remaining for even a short time in water of a lower temperature reduces the natural heat of the body, impedes the circulation of the blood, and weakens the vital powers. Cold bathing is always hazardous, and its frequent repetition is inevitably destructive of health. We refer, of course, to "still bathing," not to swimming, which is invigorating and healthful, since the exercise keeps the circulation in its normal condition. Persons who drive, and are accustomed to the care of horses, know very well that a horse may drink cold water, when he is very warm, provided he is driven briskly immediately afterward ; but if he stands still for half an hour he will "founder," or die of colic. The "cold water mania" attained its height in this country about a quarter of a century ago, and just before HALL'S JOURNAL OF HEALTH was founded for the purpose of doing battle against that and kindred evils. "Water cures" were established in many places, and became the resorts of invalids suffering from every variety of diseases, real and imaginary. We do not believe that any, whose habits had been correct and *cleanly*, were ever benefited by cold baths, while those of delicate constitutions seldom recovered from the shock of the treatment. It would make one shudder to realize the number of infants who have had their lives drenched out of them with "cold water" by their fond mothers. The most scornful look ever bestowed upon us, to our knowledge, was by a mother, in response to our protest against bathing her infant daily in cold water. She never forgave us until *after the funeral.* It is only within two or three years that our best surgeons have abandoned the use of cold water dressings in cases of fracture, etc., as not only useless, but positively dangerous.

We have seen gangrene result from allowing a small stream of cold water to trickle continuously for hours, or days, upon a fractured limb. A gentleman recently called to consult us about his ankle, which had been sprained six weeks before. He had bathed it two or three times a day in cold water during the time. It seemed to get no better, and there was a sensation as of some hard substance in the joint. We directed him to get into a bath-tub just before bed-time and let the contents of the boiler, as hot as he could bear it, flow in a small stream on his ankle. He did so, and then went to bed. In the morning he found all soreness had disappeared, and there has been no return of the trouble. We wish it understood, then, that where others use cold water, we advise the use of warm or hot water. Prof. Frank H. Hamilton, surgeon at Bellevue Hospital, has published a pamphlet on the uses of hot water in surgery.

We recently visited the hospital with him, and had our most favorable opinion of the uses of hot water in surgery confirmed. We found that the hot water dressing was not only the best ever devised for recent flesh wounds, but its effect on chronic ulcerations was marvelous.

We do not believe that frequent warm baths will result in anything but good to persons using them properly. If the temperature of the water is below that of the body, a chill may result, which always portends danger. If the temperature is much higher than that of the body, the bath, long continued, debilitates, and the bather is liable to take cold on coming out. For careless people the sponge-bath is the safest.

To derive the greatest advantage from bathing, soap should always be used, as water alone will not thoroughly remove the oily substance which the skin secretes.

